# Feasibility of a Multi-Laboratory Model of Middle Cerebral Artery Thromboembolic Stroke with Thrombolysis: TE-MCAo

**DOI:** 10.1007/s12975-025-01407-4

**Published:** 2026-01-30

**Authors:** Michelle Lin, Mozammel Bhuiyan, Carly McCurry, Jessica Lamb, Marcio A. Diniz, Karni Bedirian, Anil K. Chauhan, Abhishek Jha, Aditi Jain, Enrique C. Leira, Rakeshkumar Patel, Mohammad B. Khan, Pradip Kamat, David C. Hess, Krishnan Dhandapani, Huaxin Sheng, Sasha Zhang, Wei Yang, Bingren Hu, Chunli Liu, Lauren Sansing, Pinar Caglayan, Qinyue Guan, Ligia Boisserand, Cenk Ayata, Takahiko Imai, Kirsten Lynch, Patrick Lyden

**Affiliations:** 1 Department of Neurosurgery, Keck School of Medicine of USC, Los Angeles, CA USA; 2 Department of Physiology and Neuroscience, Zilkha Neurogenetic Institute of the Keck School of Medicine of USC, Los Angeles, CA USA; 3 Department of Population Health Science and Policy, Icahn School of Medicine at Mount Sinai, New York, NY USA; 4 Department of Internal Medicine, Division of Hematology, Oncology and Blood & Marrow Transplantation, Carver College of Medicine, University of Iowa, Iowa City, IA USA; 5 Departments of Neurology & Neurosurgery, Department of Epidemiology, College of Public Health, Carver College of Medicine, University of Iowa, Iowa City, IA USA; 6 The Medical College of Georgia at Augusta University, Augusta, GA USA; 7 Department of Anesthesiology, Duke University, Durham, NC USA; 8 Department of Emergency Medicine and Neurosciences, University of California San Diego, San Diego, CA USA; 9 Department of Neurology, Yale University School of Medicine, New Haven, CT USA; 10 Massachusetts General Hospital, Boston, MA USA; 11 Laboratory of NeuroImaging, USC Mark and Mary Stevens Neuroimaging and Informatics, Institute of the Keck School of Medicine of USC, Los Angeles, CA USA; 12 Department of Neurology, Keck School of Medicine of USC, Los Angeles, CA USA; 13 1501 San Pablo Street Room ZNI 245, Los Angeles, CA 90033 USA

**Keywords:** Cerebral ischemia, Thromboemboli, Animal model, Rigor, 3 rs

## Abstract

No single animal stroke model satisfies all needs of translational stroke investigation. While the nylon filament model is widely accepted in preclinical translational stroke research, thromboembolic models have more physiological relevance. Thromboembolic models are technically difficult, time consuming, and show variable results, making them problematic for multi-laboratory preclinical network use. We sought to develop and validate a multi-laboratory thromboembolic middle cerebral artery occlusion model that encompasses vessel occlusion and subsequent thrombolysis. To reduce the numbers of donor animals used, we developed a method to store donor blood for later use. Using prefabricated microcatheters, we simplified thrombus preparation and handling. Emboli were prepared in microcatheters and injected directly into the middle cerebral artery from the internal carotid artery. For thrombolysis we used intravenous Tenecteplase dosing at 1.5 mg/kg. To demonstrate feasibility and ease-of-use, the model was implemented at six research laboratories. We wrote and field-tested standard operating procedures, training videos, and hands-on surgical training workshops. We enrolled 170 Sprague Dawley rats of both sexes at six laboratories who performed 4 to 6 thrombus embolizations per week. All sites could achieve reproducible occlusion and thrombolysis. Thromboemboli prepared from stored blood served as well as emboli from freshly drawn blood. Of 135 rats who received one embolus, 33 (24%) died before 48-hour MRI scan. In survivors, stroke volume was 13 ± 16% of the ipsilateral hemisphere. Occlusions were seen in the MCA in 15%, distal ICA in 19%, both (T-occlusions) in 2% and in the MCA/ACA in 1%. Corner test, neurobehavioral battery, and MRI showed reasonably consistent stroke injury. We established the feasibility and reproducibility of a multi-laboratory rodent thromboembolic model but further development is needed to improve the success rate and lower the mortality rate before this model can be accepted widely.

**Supplementary Information:**

The online version contains supplementary material available at 10.1007/s12975-025-01407-4.

## Introduction

Stroke is the third leading cause of death worldwide [1]. The incidence of stroke combined with high disease morbidity results in a sizable public health impact. Despite a large body of literature identifying promising cerebroprotectants in the pre-clinical setting, none have succeeded in human trials and clinical practice.

Researchers have posited that insufficient rigor in pre-clinical models contributes to this lack of translatability [2, 3]. To address this incongruity between promising pre-clinical study results contrasted to an absence of efficacy in clinical trials, several groups around the globe have begun to implement multi-laboratory preclinical trials, patterned after multicenter clinical trials [4–7]. Multi-laboratory studies allow for the highest possible rigor including centralized blinding, randomization, and power. In 2019 the National Institutes of Neurological Disorders and Stroke initiated a multi-center consortium known as Stroke Preclinical Assessment Network (SPAN) [4]. SPAN aims to screen potential therapeutics more effectively by minimizing bias through centralized masking, randomization, and automated centralized outcome analysis. In addition, SPAN has embraced heterogeneity across multiple laboratories, thereby mimicking human trials more realistically [8]. Study drugs are blinded, randomized, and placebo-controlled at a central coordinating center, then distributed to participating research laboratories nationwide. Rodent stroke surgeries are conducted at the research laboratories with strict adherence to standardized operating protocols. Data is uploaded to a central registry for blinded and agnostic analyses.

SPAN 1 employed the most frequently utilized rodent ischemic stroke model, the monofilament occlusion of the middle cerebral artery (MCA) [9]. While the monofilament model mimics a large vessel occlusion (LVO) followed by complete reperfusion due to mechanical thrombectomy, it fails to recapitulate the etiology and biology of clinical strokes, and results in non-physiological endothelial damage. Most ischemic strokes are either cardioembolic in origin or secondary to atherosclerosis (in situ atherosclerotic plaque rupture or athero-emboli from systemic disease) [10, 11]. Arguably, the inflammatory cascade associated with such a thrombotic occlusion may be insufficiently represented by a biologically inert nylon monofilament. Furthermore, the monofilament model presupposes that all patients will experience a Thrombolysis in Cerebral Infarction (TICI) grade 3 (complete) reperfusion, which is not consistent with clinical experience. In randomized trials and registries, only a quarter of patients with LVOs will recanalize with systemic thrombolysis [12, 13]. Moreover, even with contemporary interhospital transfer capabilities, only a minority of eligible patients will arrive at an endovascular-capable facility in time to undergo mechanical thrombectomy [14]. In countries with fewer appropriate facilities, the thrombectomy rate would be even lower. An embolic thrombus followed by systemic thrombolytic administration might better represent the pathophysiology and sequence of events observed in most clinical scenarios.

To date, rodent thromboembolic stroke models have been hindered by their complexity and thus are notoriously difficult to reproduce [15–17]. These challenges have led to a disproportionate utilization of the monofilament model over embolic thrombus models. Herein, we describe our initial experience developing, validating, and implementing an updated, simplified, and standardized model of thromboembolic middle cerebral artery occlusion (TE-MCAo) and systemic thrombolysis in the SPAN network. We sought to refine the method to allow the high-throughput, high-volume studies needed in pre-clinical network trials such as SPAN.

## Methods

### SPAN Network

The SPAN Network design and basic structure have been fully described [4] and all SPAN Standard Operating Procedures (SOPs) are available at www.spannetwork.org. In brief, the network is comprised of a single coordinating center (CC) that is responsible for drug packaging and distribution, data quality control, verification of protocol adherence, and network communication. Following the acquisition of pilot data, SOPs and an experimental protocol are drafted by the CC and proposed to the Steering Committee for review. The SPAN Steering Committee is led by the CC and includes the principal investigators of each SPAN research laboratory, and NINDS staff.

**Fig. 1:**
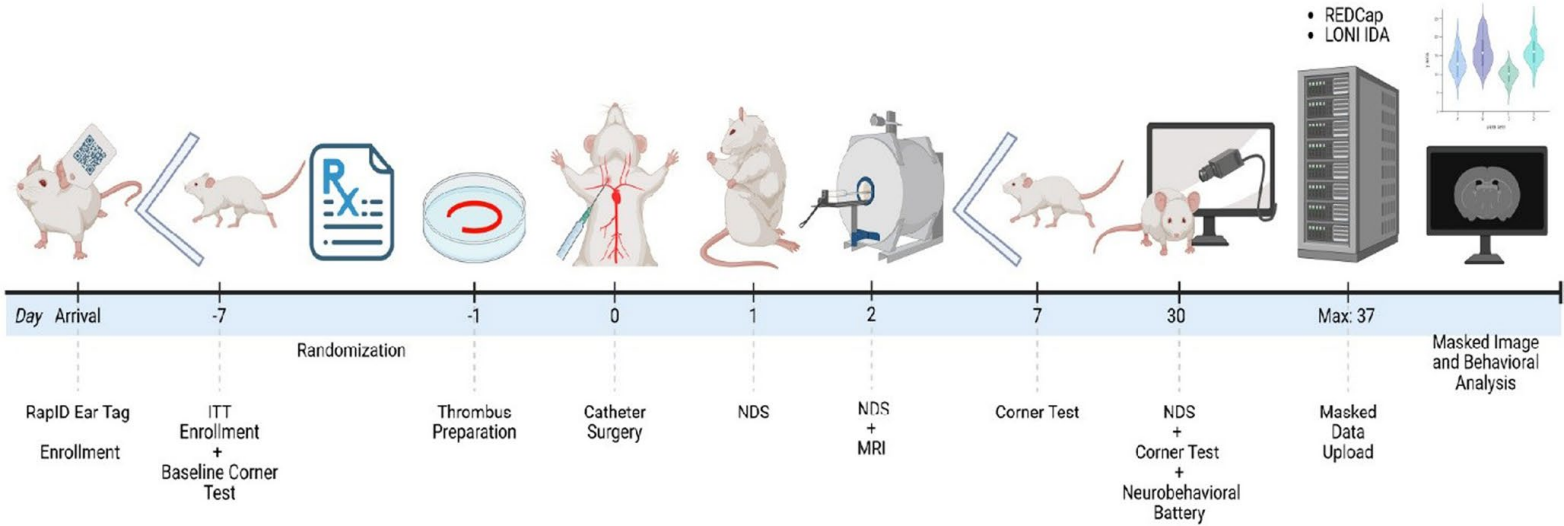
SPAN TE-MCAo Workflow. All subjects are enrolled on arrival in the research laboratory. When ready for surgery, the lab submits an intention to treat (ITT) form to the Coordinating Center. After randomization, thrombi are prepared for catheter injection on the day of stroke surgery. Follow up studies occur until 30± 5 days. Abbreviations: IDA Imaging Digital Archive; ITT Intention to treat; LONI Laboratory of NeuroImaging; MRI magnetic resonance imaging; NDS neurological deficit scale; REDCap Research Electronic Data Capture

### Subject Identification, Randomization, and Data Recording

The overall workflow for this experiment is detailed in Fig. 1. Male and female Sprague-Dawley rats (2–3 months old) in equal sex distribution were enrolled in the study. All rodents had an MRI compatible bar-coded ear tag (RapID^®^, San Francisco, CA) placed upon arrival at the research laboratories. To eliminate attrition bias, bar code numbers were entered in the SPAN Research Electronic Data Capture (REDCap) Database as each subject was enrolled [18]. One week prior to planned surgery, sites submitted an intention to treat (ITT) form to the CC for treatment randomization. The CC completed randomization using randomization tables stratified for site and sex. Animals used for donor blood collection were excluded from the ITT group.

### Therapeutics Assessed

Five putative therapeutics were investigated in this study: BPN-27,332, a lipoxygenase inhibitor; uric acid, a potent natural scavenger of free radicals; tatCN19o, a peptide inhibitor of activated Ca^2+^/CaM-dependent protein kinase II (CaMKII); GSK2256294, an inhibitor of soluble epoxide hydrolase (sEH); and NVX-058 an oxygen carrying compound. Two control groups were also included, one for the 4 intravenously (IV) administered drugs and another for the one intraperitoneal (IP) drug.

All test drugs were packaged in identical appearing vials, labeled at the coordinating center, and shipped to research laboratories at the start of the project. Tenecteplase (TNK, 1.5 mg/mL, Genentech, San Francisco) was purchased and used for inducing systemic thrombolysis. Individual 50 mg vials reconstituted at the CC, aliquoted into 2 ml vials, frozen at −20 °C, labeled, packaged, and shipped to the research laboratories. Stability of reconstituted TNK stored at −20 °C is 60 days [19].

### Blood Collection and Storage

**Fig. 2:**
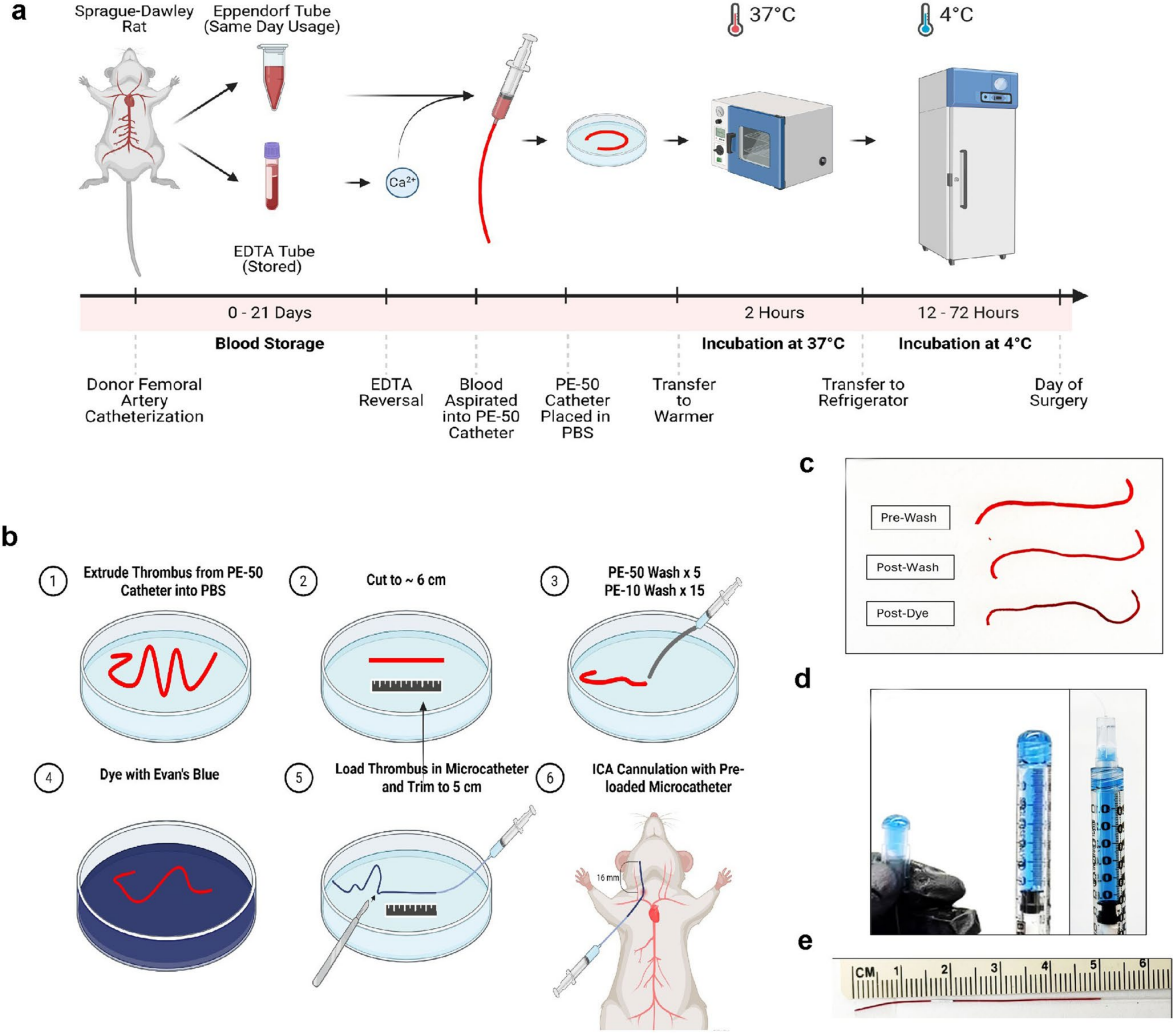
Thrombo-embolus preparation. Details are provided in text. Abbreviations: EDTA ethylenediamine tetra-acetic acid; PBS phosphate buffered saline; ICA internal carotid artery. Modified with permission from Bhuiyan et al., 2025

Femoral artery catheterization for blood collection was performed under 1–2% isoflurane with 30:70 O_2_:N_2_O mixture after induction with 4% isoflurane (Fig. 2). Donors were female or male to allow sex-matching when embolizing. A blunt-beveled PE-50 catheter was introduced into the artery and secured following brisk return of arterial blood. On average, four to six aliquots (500 µL per aliquot) of blood could be obtained from unilateral femoral artery catheterization. Aliquots were either collected into 1.5 mL Eppendorf tubes for same day use or stored for later use in Pediatric EDTA (Ethylene-diamine-tetra-acetic acid) tubes (BD Microtainer K2E – 363706). Filled EDTA tubes were stored at 4 °C for up to three weeks (Fig. 2a). Arterial blood donors were returned to the vivarium following recovery from anesthesia if unilateral catheterization had been performed. Following bilateral femoral artery catheterization, however, donors were euthanized in line with IACUC regulations.

### Thrombus Preparation and Microcatheter Loading

If stored blood was used, EDTA anticoagulant was reversed by adding 1 mg (5 mg/ml solution) of CaCl_2_ to 500 µL of blood and gently mixing. To prepare thrombi, fresh or reversed/stored blood was immediately drawn into a 100 cm of PE-50 tubing and incubated in pre-warmed phosphate buffered saline (PBS) at 37 °C for 2 h in a table-top oven to facilitate thrombosis. Then the thrombus-filled PE-50 tubing was transferred to 4 °C for storage until use, between 20 and 72 h. On the morning of TE-MCAo surgery, thrombus was expelled from one of the stored PE-50 tubes using saline flush (Fig. 2b). The 100 cm thrombus unfurled into a petri dish containing PBS. Sections of thrombus approximately 6 cm in length were cut with a razor blade (Fig. 2b). Each section was washed in saline by aspirating it into a PE-50 catheter and fully expelling it 5 times. These washes were followed by 15 washes using a smaller diameter PE-10 catheter. Complete aspiration and expulsion between each wash yields a fibrin-rich thrombus (Fig. 2c). To allow visualization of injected thrombi, the thrombus segments were dyed in 0.08% Evan’s Blue in PBS.

A 1 mL syringe with a male Luer lock tip was filled with saline, air bubbles were carefully expelled and then inverted and connected to the female Luer of the microcatheter (PE-SC1-360-200−1500, Doccol^®^ Corporation, Sharon, MA) while continuously expelling saline to create a wet-to-wet connection without air emboli (Fig. 2d). One prepared thrombus was aspirated into a microcatheter while submerged in saline. Thrombus longer than 5 cm (measured intra-catheter) was extruded and trimmed to size with a razor blade (Fig. 2b). The microcatheter was marked at 16 mm from the tip, to demarcate the extent of microcatheter that should be advanced from the bifurcation of the common carotid artery (Fig. 2e).

### Animal Surgeries

Institutional animal care and use committee (IACUC) approval was obtained at each of the research laboratories. Surgeons from all research laboratories completed standardized in-person training at the CC prior to implementation at their respective sites. Analgesia with carprofen 4 mg/kg subcutaneously and atropine 0.04 mg/kg intraperitoneally were administered to maintain animal comfort intraoperatively. Animals were induced and maintained using isoflurane as above; temperature was maintained at 36.5 °C throughout the surgery using a heating pad controlled with a rectal temperature probe.

### MCAo (Middle Cerebral Artery occlusion) and Systemic Thrombolysis

Via a ventral midline neck incision, the right common carotid artery (CCA), external carotid artery (ECA), and internal carotid artery (ICA) were identified and retracted. An arteriotomy was made in the ECA (Zea Longa CCA open methodology [9]). A microcatheter with pre-loaded thrombus was advanced intracranially to 16 mm from the carotid bifurcation under direct visualization to avoid inadvertent cannulation of the extracranial pterygopalatine artery. The intraluminal microcatheter was secured to the ICA and CCA with a single 4 − 0 silk braided ligature. Injection of the 1 mL syringe was performed with a motorized syringe pump (Braintree Scientific BS-300 Syringe Pump), at a rate of 10 µL/minute for 2 min, followed by an additional 20 µL of sterile saline over 2 min. The microcatheter was subsequently removed and the ECA was permanently ligated with a suture proximal to the arteriotomy site. The skin incision was temporarily closed (staples or sutures) and the animal awakened on a heating pad. After re-anesthesia, systemic thrombolytic was infused 2 h after thrombus injection with 1.5 mg/kg of intravenous Tenecteplase (TNK) via an external jugular vein. Following TNK, the animals recovered and when fully awake returned to the vivarium. Impaired animals received subcutaneous saline, 1 ml/100 g, twice daily until they were freely drinking.

### Cerebral Perfusion Imaging

Pilot studies were conducted to evaluate intraoperative cerebral blood flow in response to thrombo-embolism and systemic thrombolysis. These pilot studies were used to guide model development and subjects were not included in the total enrolled subjects. Thrombi were prepared and pre-loaded into microcatheters as above. A microcatheter was secured in place with 4 − 0 silk ligatures at the ICA and CCA, as well as a retention ligature into the adjacent omohyoid muscle. A superficial external jugular vein was cannulated with another microcatheter and filled with saline. After assuring both the arterial and venous catheters were secure, the skin incision was closed with 5− 0 Prolene. The rodent was then repositioned prone under continuous anesthesia. A midline incision was made over the cranium, the pericranium and temporalis were dissected from the underlying periosteum. A rotary drill was used to thin the calvarium until the cerebral cortical vasculature was well visualized on laser speckle (RFLSI-ZW Laser Speckle Contrast Imaging System, RWD Corporation, Shenzhen, China). Baseline hemispheric perfusion was recorded bilaterally by selecting regions of interest in the distribution of the MCA territory. After baseline cerebral blood flow recording, a thrombus was injected through the pre-positioned arterial catheter and post-injection perfusion was monitored. This was followed 30 min later by systemic thrombolysis (1.5 mg/kg of TNK) via the venous catheter. Cerebral perfusion was monitored continuously for 2 h following which subjects were euthanized. Brains were immediately removed and immersed in 4% paraformaldehyde for 48 h, then transferred to 30 mg/dl sucrose until sunk, and then stored in PBS with 0.05% sodium azide. After suitable fixation, each brain was photographed from a ventral perspective and then sectioned into 1.0 mm thick slabs with a brain slicer (Harvard Apparatus, Cambridge) and photographed.

### Behavioral Assessment

A baseline corner test was obtained for all subjects one week prior to intended surgery date and repeated on postoperative days 7 and 30 [4]. Subjects were placed facing a walled apparatus (30° angle) and 10 trials were performed. Video recording was obtained, de-identified, and analyzed using Deep Lab Cut for automated turn detection [20]. The corner test index was defined as

$$\:abs\left[\frac{left\:turns-right\:turns}{left\:turns+right\:turns}\right]$$.

A higher corner index correlated with greater asymmetry in directionality of turns.

A modified Bederson neurologic evaluation (NeuroDeficit Score, NDS) was performed on postoperative day 1, 2, and 30 [21]. Animals were observed for normal behavior (0 points), or one point was scored for any asymmetric forelimb and torso turning, circling, inability to bear weight, or absence of movement.

The SPAN Neuroscore Battery, consisting of 9 behavioral assessments, was performed on postoperative day 30. The following functions were evaluated on a scale of 0–3 with 3 being the most impaired: spontaneous activity, circling, limb movement symmetry, forepaw outstretching, body and trunk sensation, vibrissae sensation, face sensation, beam walking (with a goal of 5 min), and grid climbing (with an objective of crossing within 1 min). A composite score ranging from 0 to 27 was generated from the summation of all neurobehavioral tests.

### Imaging

Magnetic Resonance Imaging (MRI) was obtained for all subjects on postoperative day 2 under anesthesia with 1–2% isoflurane in 30:70 O_2_:N_2_O oxide. The SPAN imaging protocol included multi-echo T2-weighted and diffusion-weighted imaging (DWI) sequences (with a resolution of 150 μm in-plane and 500 μm slice thickness) [22]. Scans were acquired at the six respective research laboratories using Bruker scanners (7T, 9.4T, or 11.7T). We collected multiparametric MRI data using sequences comparable to clinical protocols for assessing acute ischemic stroke. T2 maps were generated using two different methods depending on the hardware. For volume coil sites, we employed a multi-slice multi-echo sequence (TR = 4500 ms, 10 echoes from 10 to 100 ms). For a site with a surface coil site, we used sequential single echoes (15, 45, 75 ms) to mitigate biased signal variation along the depth direction. To derive the ADC map, a conventional spin-echo DWI pulse sequence was applied (TR/TE = 1500/25 ms) with b-values of 0, 500, and 1000 s/mm² in the z-direction. Angiograms were derived from time-of-flight (TOF) images (TR/TE = 4.285/15ms, flip angle = 80, Bandwidth 96153.8 Hz). Following acquisition, all datasets were resampled via tricubic interpolation to achieve an isotropic voxel size of 150 μm. The final image specifications for all scans were a 128 × 128 matrix, 30 slices, a 0.5 mm slice thickness, and a 19.2 mm x 15 mm field of view.

 [22]. DICOM files were uploaded into a centralized system Laboratory of Neuroimaging Data Archive (LONI IDA) at the University of Southern California (USC) [23]. Images were masked for centralized analysis.

MRI analysis was carried out using an automated processing pipeline implemented using the Quantitative Imaging Toolbox (QIT) [22, 24]. For preprocessing, we first applied adaptive non-local means denoising and then standardized the image resolution to 150 μm isotropic. We then calculated quantitative parameter maps, T2 relaxation time and Apparent Diffusion Coefficient (ADC), from the T2 and DWI scans. A deep learning model (U-net) was used for brain extraction [22, 25]. There were inter-site differences in quantitative MRI parameters, likely due to variety in imaging hardware and physiological factors, so we performed global intensity harmonization of each individual scan. Using a smoothed histogram of the brain intensity distribution, we identified the peak value (the mode) and scaled the entire image to bring the most likely value to one. We chose the mode because it is less affected by distributional skew due to lesions.

To identify the stroke lesion, we applied multiple thresholds to the processed T2 (specifically R2 = 1/T2) and ADC maps. An initial lesion area was identified using specific thresholds for R2 with inverted sigmoid > 0.8 and ADC > 1.5. This area was refined using smoothing and hysteresis thresholding (strong = 0.55, weak = 0.45) to create a lesion mask. A similar thresholding method identified cerebrospinal fluid (CSF) (R2 > 0.75, ADC < 1.25). Lesions were characterized by high R2 and low ADC signals, while CSF had low R2 and high ADC. Lesion volume fraction was then expressed as the total lesion volume divided by the ipsilateral hemisphere volume.

Improved visualization of the vasculature was first achieved through histogram intensity enhancement of TOF images. The tubular anatomy of the cerebrovasculature was then extracted using a Frangi filter using a sobel operator and a lower contrast threshold of 0.1 [26]. Binarized masks were generated from the Frangi image using a contrast-based threshold of 0.02, and a 3D mesh rendering of the vessels was extracted using the marching cubes algorithm [27]. Presence or absence of ACA, MCA or PCA segments were determined based on vessel continuity in the surface-based rendering of the angiogram, and further confirmed in the enhanced contrast TOF images.

We measured brain midline shift by first locating the brain’s center using the geometry of the lateral and third ventricles (identified via an atlas mask near the corpus callosum). We calculated the average 3D position of CSF within these ventricles to estimate the anatomical midpoint. A surface dividing the hemispheres was generated based on this midpoint. The absolute shift was determined by comparing this estimated midline to a standard atlas midline. The midline shift index was calculated as the absolute shift divided by the brain width.

### Thrombus Lysis Assay

To ensure consistency in forming thrombus from fresh or stored blood, we performed a thrombolysis assay on thrombi made from a variety of sources. Freshly collected or stored/EDTA calcium reversed, arterial or venous blood, were used to form thrombi. Thrombi were washed in PBS, cut to a uniform 1 cm length, and placed into a single well within a 24-well plate containing 1.0 ml of PBS. Thrombi were incubated with PBS or 0.75 mg/mL of TNK in PBS on an orbital shaker (50 RPM) at 37 °C for 4 h. Hemoglobin release was assayed with Drabkin’s Reagent (Sigma Aldrich MAK115-1KT) according to manufacturer’s protocol. Emission at 400 nm was detected on a spectrophotometer (Molecular Devices SpectraMax i3). Visual evaluation of thrombolysis was also documented and recorded as complete dissolution versus residual thrombus following treatment.

**Fig. 3:**
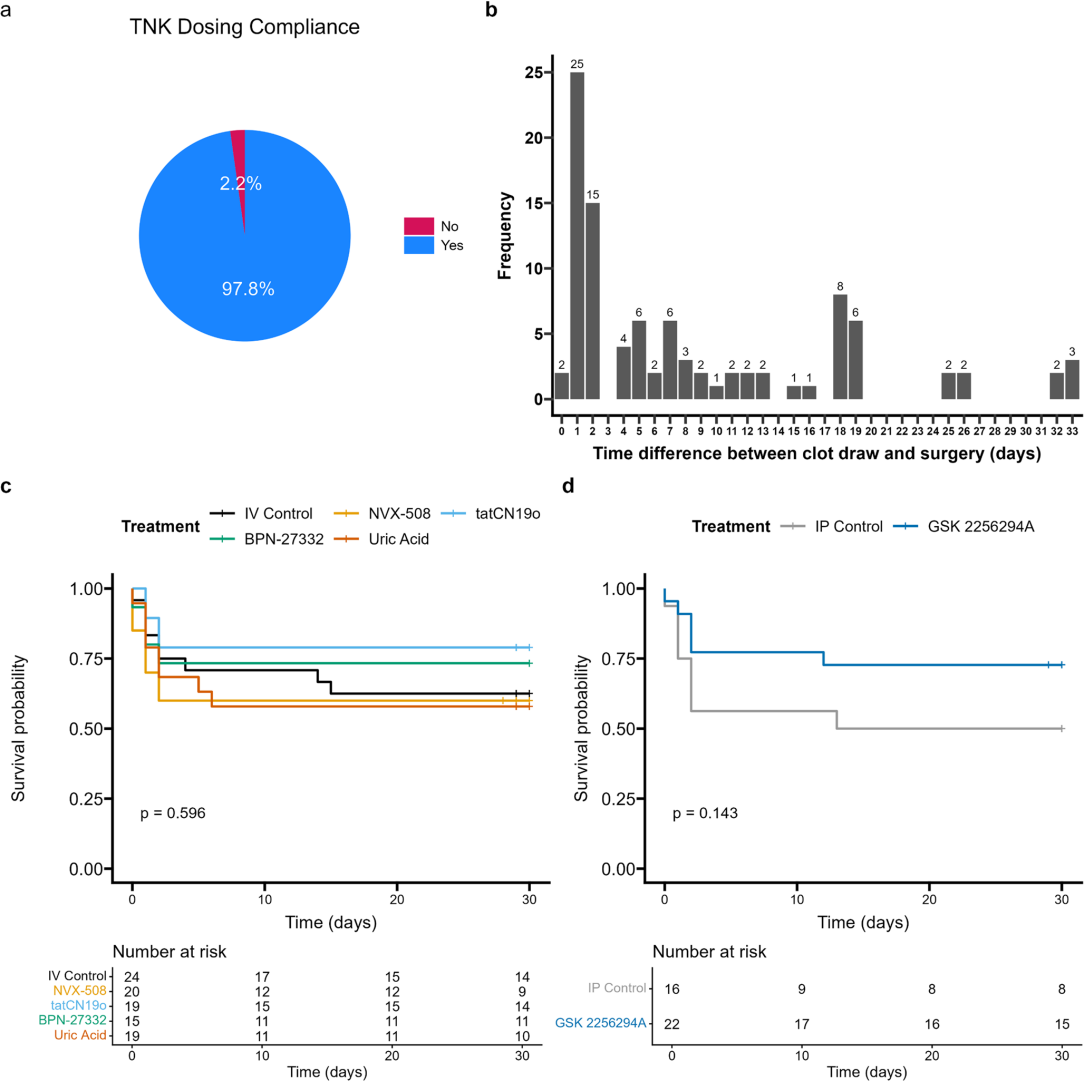
Compliance and Mortality. (**a**) Labs correctly gave TNK (correct dose, correct time) in 98% of cases. (**b**) Time from preparing thromboembolus to administration varied over 33 days. (**c**) Kaplan Meier plots showing survival for all IV treatment groups. (**d**) Kaplan Meier survival plot for one IP treatment

### Statistical Analysis

The primary analysis was defined as modified ITT population including all animals that were randomized and had successful surgery. Data were summarized with mean ± s.d. for numerical variables and displayed for numerical variables using violin plots; frequencies and bar plots were used for categorical variables, and survival curves were presented using the Kaplan-Meier method. Numerical and ordinal variables were compared between study arms (intervention vs. control) adjusted by site and sex using Probabilistic Index models, which is a generalization of the Mann-Whitney test that accommodates covariates [28]. Survival curves were compared using the log-rank test. Missing data was handled using the three approaches (1) no imputation, (2) worst score by sex and study arm and (3) multiple imputation with surgery weight, surgery age, site, sex, study arm for all endpoints and volume lesion fraction on day 2 for endpoints on day 30.

All hypotheses were two-sided, and 5% significance level was considered. No adjustment for multiplicity was applied.

## Results

### Feasibility and Protocol Adherence

For this pilot study, 170 animals were enrolled (registered) at the participating laboratories. Of these, 26 were used as blood donor animals, leaving 144 (72 male, 72 female) in the ITT group. The CC randomized subjects to one of 5 candidate treatments before the laboratory attempted MCAo. There were 9 procedural dropouts (6 died during MCAo surgery, 3 during recovery) leaving 135 animals who successfully underwent TE-MCAo surgery (modified Intention to Treat, mITT). Following surgery, 115 subjects (Fully Treated, FT) survived the initial procedure with full catheter advancement and received all treatments (thrombolysis plus randomized treatment or placebo); 20 subjects received only partial treatment before they died. After treatment, there were 29/115 (25%) subjects lost, leaving 86 subjects in the Full Analysis group. In the mITT group, TNK was administered correctly in 98% (Fig. 3a), and sex of the donor blood matched the recipient in 98%. Most thrombi were used within 48 h of donor blood draw, with an even distribution over the following 33 days (Fig. 3b). The most notable drop off in survival occurred in the first 3 days after surgery (80% survival 3 days after stroke in the mITT population), minimal death was seen between postop day 3 (78% survival) and day 30 (75% survival). Of the subjects that survived and received full treatment, the correct blinded therapeutic was administered in nearly all subjects (98.26%). For illustration of the feasibility of this model, survival curves by randomized, blinded treatment groups are shown, although no treatment effects were expected in this small pilot study (Figs. 3c, d).

The mean ± SD age of subject at the time of TE-MCAo surgery was 2.6 ± 0.3 months. The mean ± SD duration of surgery was 82 ± 18 min and 100% of subjects received anesthetic and fluids appropriately administered according to SPAN SOPs. There was exceptional compliance with surgical protocol, the mean ± SD thrombus length was 5.0 ± 0.2 cm (4.5–6.0 cm) and mean ± SD time from thromboembolism to systemic TNK administration was 121 ± 2.4, minutes (range 118–136).

### Imaging

**Fig. 4:**
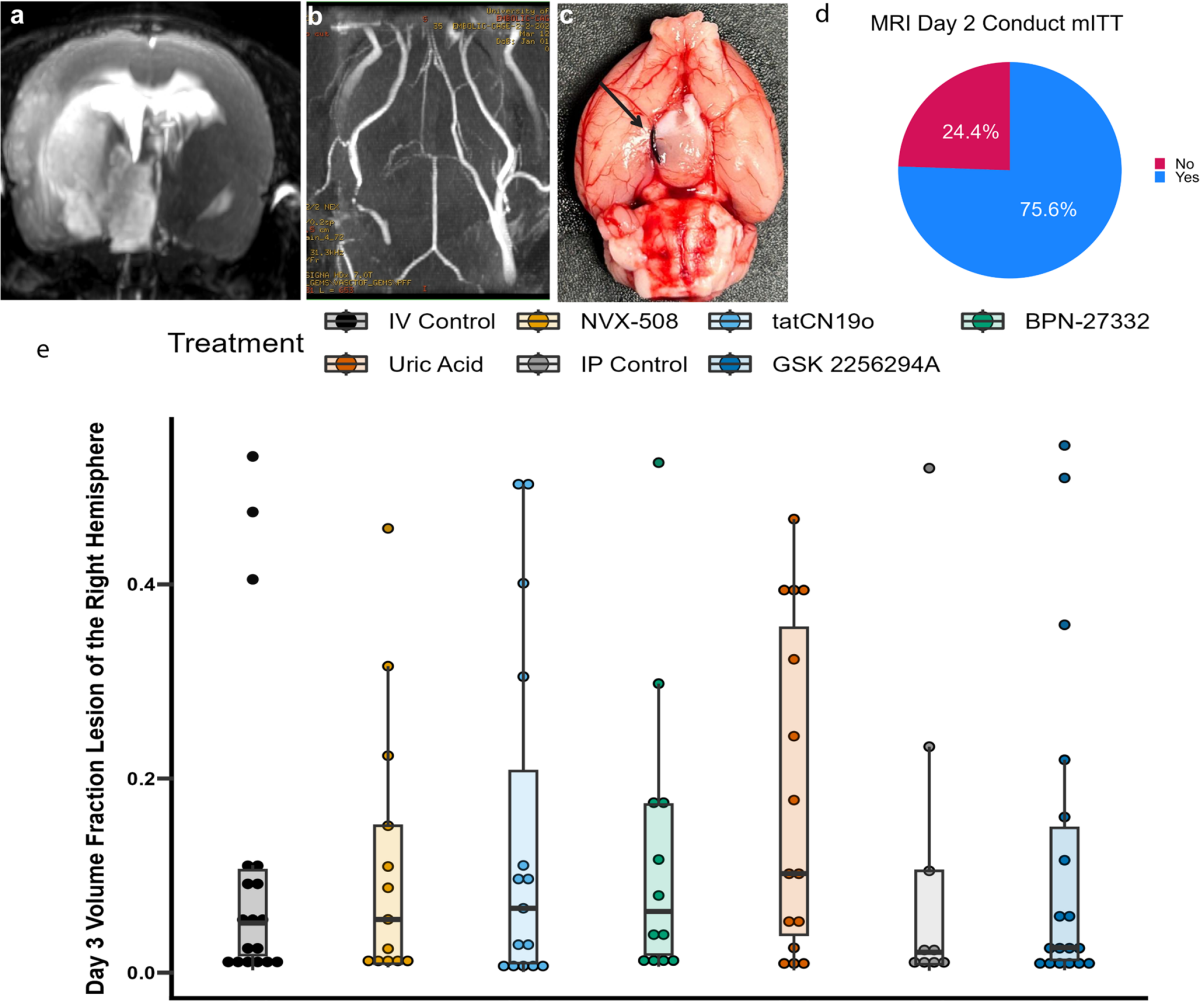
MRI. (**a**) lesion imaged with multi-echo multi-scan volumetry (**b**) Time of flight angiography showing occluded right MCA and distal ICA. (**c**) Ventral surface of brain shown in panels a and b confirming thromboembolus lodged in distal ICA. (**d**) MRI was obtained in 75% of ITT population. (**e**) Lesion volume as a fraction of the ipsilateral hemisphere by treatment group

MRI sequences included volumetry and time-of-flight angiography (Figs. 4a-c). MRI was conducted 48 h after TE-MCAo in 102/135 (75.5%) mITT subjects. Missing data was due to early mortality in 33 (24%) (Fig. 4d). Two subjects’ scans failed the automated pipeline, one due to a missing sequence and another due to poor image quality. No images demonstrated significant motion artifact, scanner malposition, failed NifTi conversion, ghosting artifact, masking threshold error, or atlas registration failure precluding pipeline processing. The mean volume fraction lesion of the right hemisphere was 13 ± 16%. For illustration, lesion fractions are shown for all coded treatment and control groups (Fig. 4e). Although there are a few outliers, overall the coefficient of variation of 130% is comparable to previously published work using this model using Sprague Dawley rats [29]. While we found no statistically significant differences in morphometry across site or by sex and volumes of ventricles, there is wide variability that will need to be studied further (Supplemental Figure). Time of Flight angiograms were omitted by the labs in 4/102 (4%) and failed the pipeline conversion in 24 (24%). No occlusion could be found in 36%. Occlusions were seen in the MCA in 15%, distal ICA in 19%, both (T-occlusions) in 2% and in the MCA/ACA in 1%.

### Behavioral Outcomes

**Fig. 5:**
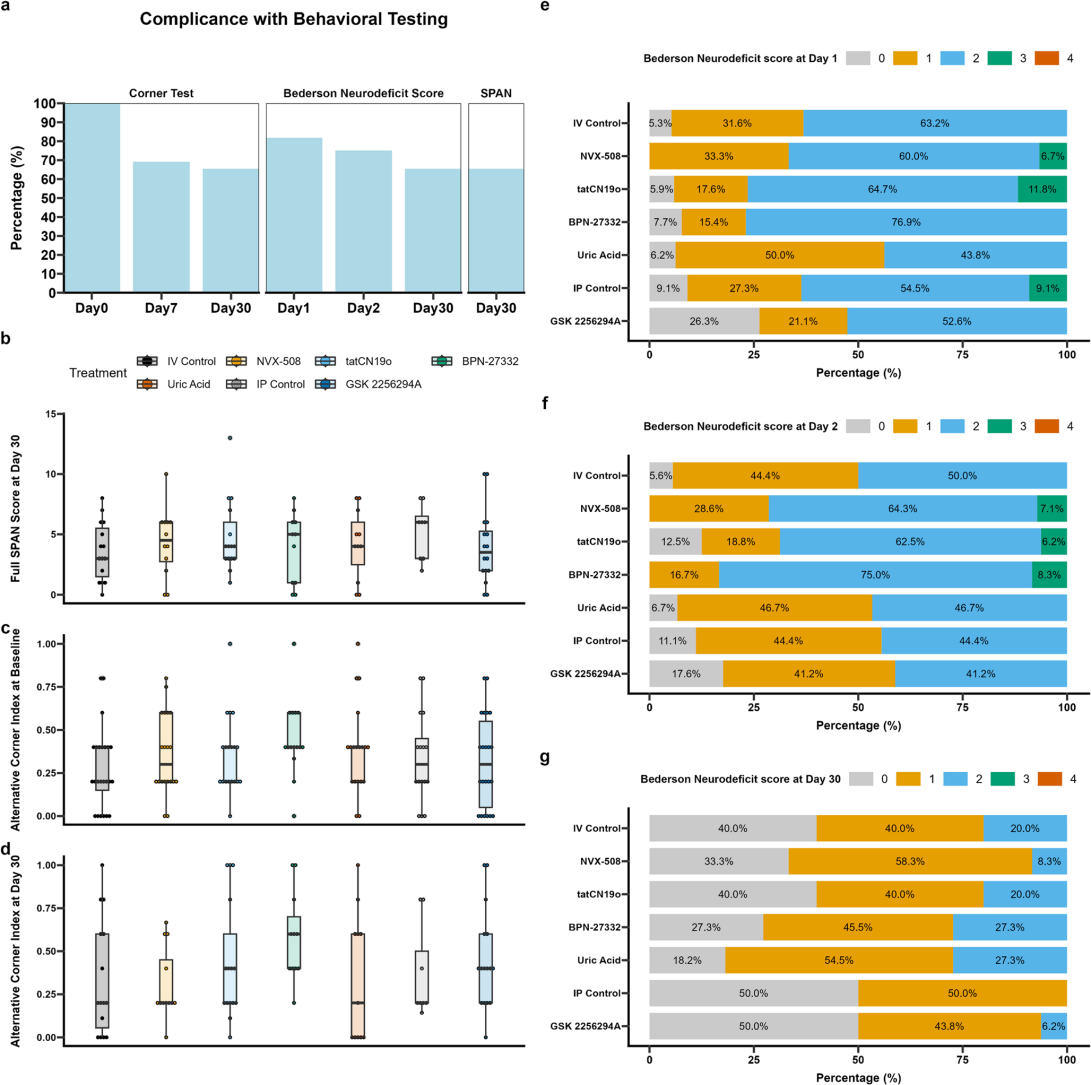
Behavior Testing Feasibility. (**a**) Corner test, NDS, and SPAN battery were completed on all surviving subjects. (**b**) Full SPAN scores showed reasonable deficits cross all groups. (**c**) Baseline Corner test by group. (d-f) NDS scores evolved over Days 1, 2 and 30 across all groups

Compliance with behavioral testing was excellent with missing data due only to subject death (Fig. 5a). A SPAN NeuroScore battery was conducted on all surviving subjects on day 30. The average score was 4.23 ± 2.77 (Range 0–13) with no differences across sites, sex, or treatment (Fig. 5b).

All mITT animals had a baseline corner test conducted, similarly all surviving mITT animals were tested at day 7 (93/135, 70%), and day 30 (88/135 65%). All missing corner tests were attributable to subject death prior to behavioral test. The mean alternative corner index at baseline was 0.35 ± 0.24, which increased to 0.52 ± 0.35 on day 7, with no differences among groups (Fig. 5c), and trended back towards baseline by day 30 when the mean was 0.40 ± 0.31, again with no significant differences among treatment groups (Fig. 5d).

Immediate post-stroke Neurodeficit Scores (NDS) were obtained in 110 of 135 (90%) of mITT subjects (Fig. 5a). Of these subjects, 10 (9%) demonstrated no deficit, with no differences across treatment, sex, or site (Fig. 5e). NDS scores were obtained on 75% of subjects on postoperative day 2, again with no differences across groups (Fig. 5f). Subjects demonstrated marked functional improvement over the course of the next month. NDS scores were obtained in all surviving mITT subjects on day 30 at which point 33 (38%) had no evidence of deficit, the remaining subjects had minor deficits, with no discernible differences across groups (Fig. 5g).

### Thrombus Lysis Assay

**Fig. 6:**
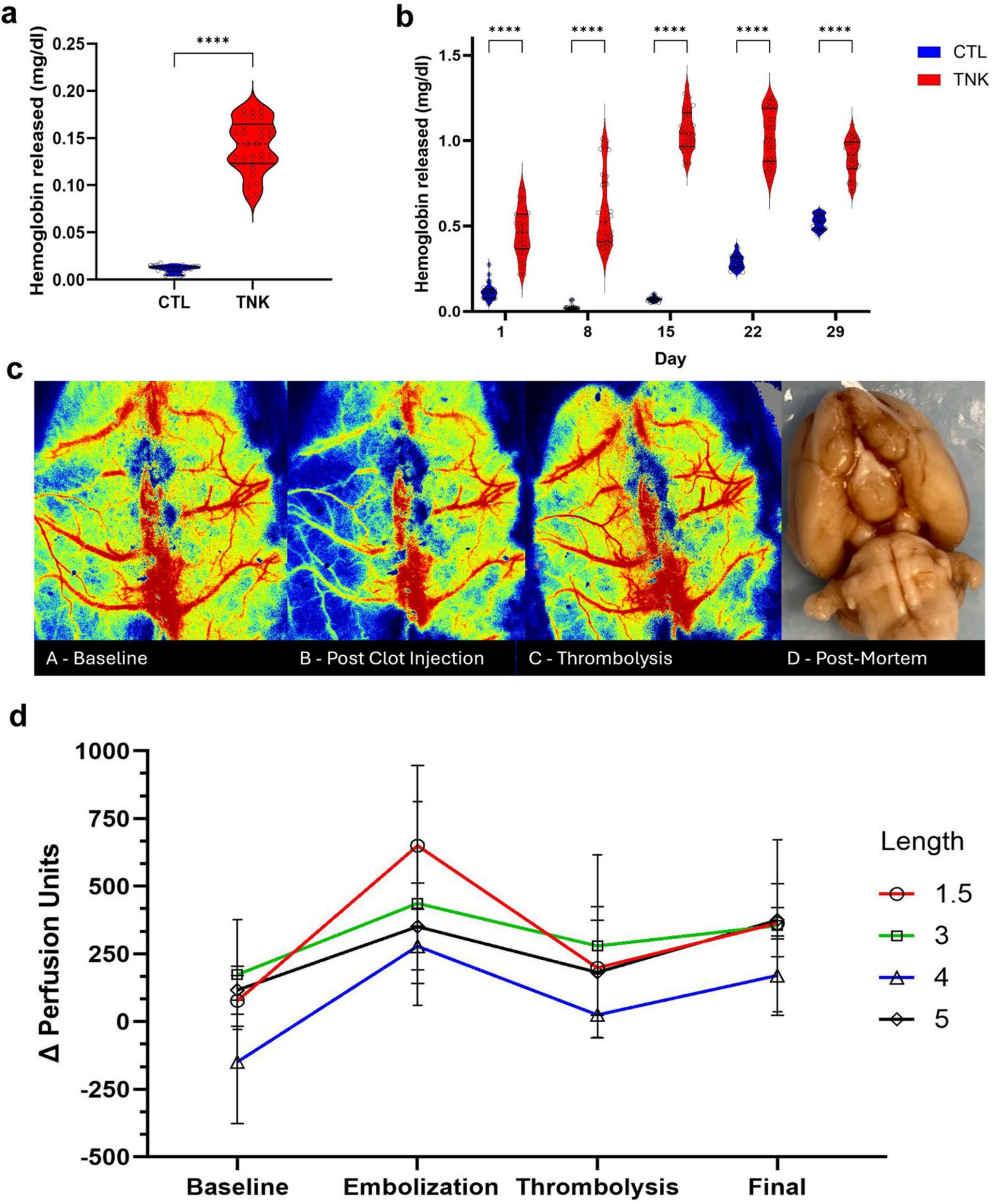
Assays for thrombolysis. (**a**) Fresh thrombus successfully lysed with TNK. ****p < 0.0001, unpaired t-test (**b**) Stored blood prepared into thrombi successfully lysed with TNK. ***p < 0.001 ANOVA (**c**) Serial laser speckle imaging of cerebral perfusion. (**d**) For a variety of lengths of thromboemboli, TNK successfully restores perfusion to baseline (*n* = 2 to 4 per group). F_2225, 27.7_=11.4 *p* < 0.001 ANOVA

Fresh thrombus from both female and male donors demonstrated a statistically significant release of hemoglobin compared to control, following treatment with TNK (Fig. 6a, t-test, *p* < 0.0001). Female blood stored in EDTA tubes and reversed with calcium chloride could be used to form thrombus following 1, 2, 3, and 4 weeks of storage (Fig. 6b). Thrombus formed from stored blood demonstrated a statistically significant (ANOVA, *p* < 0.0001) release of hemoglobin compared to control, following treatment with TNK, after 1, 2, 3, and 4 weeks of storage. However, spontaneous lysis of the control clots occurred after 22 days, suggesting the maximum useful storage time for donor blood is 15 days.

### Cerebral Perfusion

To characterize the performance of the thrombolysis in this model, injected emboli of various lengths (1.5, 3, 4, or 5 cm) were prepared as above and cerebral perfusion with laser speckle imaging was used to follow recanalization (Fig. 6c). Following thrombus injection, all subjects (*n* = 12) demonstrated a drop in cerebral perfusion (Fig. 6d). Recanalization following administration of systemic thrombolysis was observed in six (50%) subjects. We did not observe a statistically significant correlation between thrombus size and recanalization rate.

## Discussion

We developed a version of the thromboembolic rodent model of ischemic stroke (TE-MCAo) with enhancements that make it suitable for use in a multi-laboratory pre-clinical testing network (Fig. 1). This network completed multiple surgeries (> 4) per day in multiple labs simultaneously. By refining the use of stored blood, we greatly simplified the preparation of thromboemboli to allow for multiple surgeries in one day. Using stored blood also reduced the numbers of blood donor animals needed. The protocol for preparing the emboli was simplified (Fig. 2), most notably by pre-loading thrombi in pre-fabricated microcatheters to avoid custom—and variable—stretching of catheters in each case. We demonstrated feasibility across 6 different research laboratories and showed excellent protocol adherence (Figs. 3, 4 and 5). For illustration of how this model might be useful in translational research, we piloted the study of 5 different treatments currently under study in SPAN 2 and showed feasibility.

Although animal models are the foundation of pre-clinical drug testing, we seek to adhere to the 3 Rs of medical research: replace, reduce, and refine [30]. While in vitro models clarify mechanistic processes, no pharmacotherapeutic is approved for human clinical trial without robust pre-clinical mammalian studies. It is important that the preclinical model chosen is representative of the patient population the drug is intended to treat. Focal ischemic stroke models include rose Bengal photothrombotic models, cortical pial artery occlusion, the distal MCAo clip, and stereotactic endothelin-1 injection [7, 31–34]. However, intraluminal monofilament or thromboembolic models are the established models of choice for LVO [9, 35, 36].

While the nylon monofilament model is simple to execute and widely adopted, arterial blockage is induced with a biologically inert material that fails to render all aspects of the inflammatory cascade associated with release of thrombin, plasminogen, and other thrombus elements. Despite this improved fidelity of thromboembolic models over monofilament occlusion models in recapitulating stroke biology, the technical complexity of thromboembolic models and limited reproducibility has hindered their widespread utilization. A query of the Stroke-SOLES database returned 1017 studies published in 2024 using a temporary MCAo model, of which only 92 (0.09%) were coded as thromboemoblic models [37]. Since the pioneering Zhang et al. study, several labs have described their experience applying a thrombo-embolic model in rabbit, porcine, canine, and non-human primate studies [35, 38–42]. The studies are often infeasible for the standard research lab, may require fluoroscopic guidance, are prohibitively costly, and may not be in accordance with US ethical and regulatory standards. In this study, we have refined the thromboembolic model to develop a multi-laboratory rodent heterologous embolic thrombus and thrombolysis model employed within the rigorous SPAN framework. We aim to further reduce the numbers of animals needed to rigorously evaluate putative cerebroprotectants.

### Scientific Rigor of Pre-Clinical Studies

To increase the translational potential of preclinical animal models, the 11th Stroke Therapy Academic Industry Roundtable (STAIR XI) group established guidelines for scientific rigor, of which this SPAN study follows nearly all of them [43]. It is recommended that treatment effects are studied over an extended time frame: we performed periodic behavioral testing over a course of 30 days. As opposed to evaluating immediate post-stroke histology, multiple methods were employed to assess stroke severity including three distinct behavioral tests and imaging with MRI. SPAN’s centralized and masked treatment distribution minimizes allocation bias and concealment. Surgeries were conducted with rodents of both biologic sexes in equal proportion. Outcome data was uploaded to a centralized database for quality control, followed by masked behavior analyses or automated image processing. Furthermore, time points for intervention were chosen in a clinically relevant fashion - thrombolysis was administered 2 h after thromboembolism, and study drug treatments after that. Two hours approximates the mid-point for when patients are still candidates for systemic thrombolysis, 4.5 h from last known well time.

### SPAN Thromboembolism Model

The first description of a rodent thromboembolic model that we could find was published by Kudo et al. in 1928 [44]. The authors performed an intracardiac puncture to harvest arterial blood, which was incubated for 48 h at room temperature, fragmented into thrombi under 100 μm in size, and injected as a suspension of microemboli. Despite this early publication, a thromboembolic model did not reach the scientific zeitgeist until the 1990’s when Overgaard et al. published a method with microemboli suspension [45] and Zhang et al. described a novel model embolizing a 25 mm homologous thrombus [35]. A variation of this protocol detailed by Zhang et al. continues to be the most frequently employed rodent model of thromboembolic large vessel occlusions [46]. Microemboli in rabbits were used to first demonstrate the effect of alteplase on neurological function [47].

While many earlier models utilized an intracardiac puncture for harvest or arterial blood, we employed a femoral artery catheterization. Combining this with blood storage, we were able to produce thromboemboli for eight to ten sets of surgeries from a single donor, reducing the number of allogenic donors needed. Using a thrombus lysis assay, we found that both freshly drawn female and male, as well as blood stored for up to four weeks, responded to TNK treatment (Fig. 6). The rate of spontaneous lysis in the stored blood increases after 2 weeks however, which is the longest useful storage life. Additionally, we were able to produce sizable intact thrombi without thrombin or fibrin augmentation which has been demonstrated to increase thrombus fracture rates [48].

Most previous studies catheterized the carotid artery with a modified PE-50 catheter. Following thrombus loading into the PE-50 catheter, heat modification is required to taper the tip for navigation into the small luminal caliber of the intracranial circulation. The added complexity of this heat modification introduces potential variability in catheter preparation and increases the chances of inadvertently introducing air into the catheter. Our protocol of sequential washing with progressively smaller caliber tubing created a thrombus compatible with direct loading into a pre-fabricated microcatheter, but still of sufficient size to create consistent LVO infarcts. We utilized a microcatheter with a Luer lock attachment that did not require post-market customization. This facilitated catheter priming without risk of air embolus, increasing the efficacy of thrombolytic therapy. Surgeons across all study sites reported satisfactory carotid catheterization and navigability with this microcatheter. The use of a mechanical syringe pump further minimized variability in velocity and force across procedures, as prior studies have found rate of injection to significantly impact stroke size [17].

### Alternative Models of LVO

In recent years, two additional models have gained traction for studying acute focal ischemic stroke in rodents; these are the endothelin-1 application or thrombin injection models. Endothelin-1 is a potent vasoconstrictor that can simulate a temporary MCAo. Initial models stereotaxically injected endothelin-1 into the striatal region to mimic a lacunar stroke [49], but this is increasingly being applied to cortical MCA vessels [33, 50]. The endothlin-1 model lacks the intraluminal perturbation seen in monofilament and thromboembolic models. More importantly, the strokes tend to be smaller in size, reperfusion occurs gradually as vasoconstriction resolves, and behavior deficits are less consistent [51]. Alternatively, some labs have reported a thromboembolic model wherein thrombin is preloaded into a catheter that is placed in the ICA, blood is then withdrawn and allowed to coagulate within the catheter, which is subsequently injected into the intracranial circulation [52–54]. One limitation of this model is the potential inconsistency in thrombus length injected. Additionally, studies have reported thrombin admixed thrombi to be resistant to systemic thrombolysis [55]. Niessen et al. in 2003 compared autologous spontaneously forming thrombi with thrombin augmented thrombi and found crucial differences in elasticity and disintegration [55]. Most notably, blood perfusion and apparent diffusion coefficient returned to baseline following thrombolysis in the spontaneously formed thrombi but not in those that were mixed with thrombin [55].

## Limitations

A notable limitation of our study is the high mortality rate experienced in our index series, 25% of our effective sample size. While this rate is comparable to a 90-day mortality rate after LVO of 26% in human studies, such a high early mortality rate raises complex issues for handling missing data [56]. At the same time, we also had a high rate of lesion volume close to zero, that is, a failure to cause occlusion (Fig. 4). Together, these two problems render the model much less desirable. We are currently investigating whether different sized thrombi would lead to a lower peri-operative mortality rate, increasing our effective sample size, while also raising the rate of successful occlusion. While limited by insufficient sample size, we have demonstrated a quantifiable decrement in ipsilateral perfusion following thrombus injection with thrombi as small as 1.5 cm in our pilot studies. Further research will be needed to identify the optimal thrombus length. Another limitation is our failure to visualize intracranial occlusion in 36% of animals that suffered measurable stroke. We suspect these were extracranial ICA occlusions and further work is needed to improve our ToF methods.

We did not perform continuous physiological monitoring of the animals during MCAo surgery. Such monitoring is often touted as necessary to assure better lesion reproducibility. Physiological monitoring, however, requires invasive instrumentation that requires more time than the MCAo surgery itself. Therefore, in SPAN we embrace heterogeneity [4, 8], which is a characteristic of human stroke, and avoid invasive physiological monitoring.

This model has room for improvement. While we were able to successfully implement this model with consistency across six centers, some surgeons noted the thrombus preparation to be cumbersome. To this end, we are investigating whether less washes can be performed, to decrease thrombus preparation time. Additionally, a longer microcatheter (Doccol^®^ PE-5 L-360-200−300) is also being incorporated into our workflow to improve ergonomics of using the mechanical syringe pump. Another major limitation is the use of healthy young rats in our study. This was primarily intended to be a pilot study on the feasibility of implementing a thromboembolic model across multiple laboratories, therefore we opted to study healthy young subjects. However, future studies would undoubtedly be improved by the incorporation of aged and hypertensive rats as performed in SPAN1 [57]. Morphometry—lesion and ventricle volumes, and midline shift—varied across sites (Supplemental Figure S2). The sample sizes are small and include the presence of a “learning curve” effect.

## Conclusions

Rodent stroke models underpin preclinical translational research. While an intraluminal embolic thrombus followed by systemic thrombolysis best recapitulates the most frequently observed clinical scenario of a large vessel occlusion, thromboembolic models have historically been limited by technical difficulty, excessive variability, and limited reproducibility. We present our initial experiences implementing a thromboembolic rodent model across multiple laboratories and operators with high fidelity, but we have yet to derive a model that is ready for wider deployment.

**Table:** 

Mortality - Site						
Variable	Site					
	AG (*n* = 23)	DK (*n* = 30)	IW (*n* = 11)	MG (*n* = 28)	SD (*n* = 24)	YL (*n* = 19)
Animal Death at Day 3						
No	19 (82.61)	22 (73.33)	9 (81.82)	23 (82.14)	10 (41.67)	12 (63.16)
Yes	4 (17.39)	8 (26.67)	2 (18.18)	5 (17.86)	14 (58.33)	7 (36.84)
Effective Sample Size	23	30	11	28	24	19
Animal Death at Day 7						
No	18 (78.26)	22 (73.33)	9 (81.82)	23 (82.14)	9 (37.50)	11 (57.89)
Yes	5 (21.74)	8 (26.67)	2 (18.18)	5 (17.86)	15 (62.50)	8 (42.11)
Effective Sample Size	23	30	11	28	24	19
Animal Death at Day 30						
No	7 (30.43)	8 (26.67)	4 (36.36)	23 (82.14)	8 (33.33)	7 (36.84)
Yes	16 (69.57)	22 (73.33)	7 (63.64)	5 (17.86)	16 (66.67)	12 (63.16)
Effective Sample Size	23	30	11	28	24	19

## Supplementary Information

Below is the link to the electronic supplementary material.

Supplementary Material 1 (JPG 452 KB)

## Data Availability

No datasets were generated or analysed during the current study.
