# Supplementary figures and images for: Feasibility of a Multi-Laboratory Model of Middle Cerebral Artery Thromboembolic Stroke with Thrombolysis: TE-MCAo
Source: Transl Stroke Res. 2026 Jan 30;17(1):20. doi: 10.1007/s12975-025-01407-4 (PMC12855246; doi:10.1007/s12975-025-01407-4)

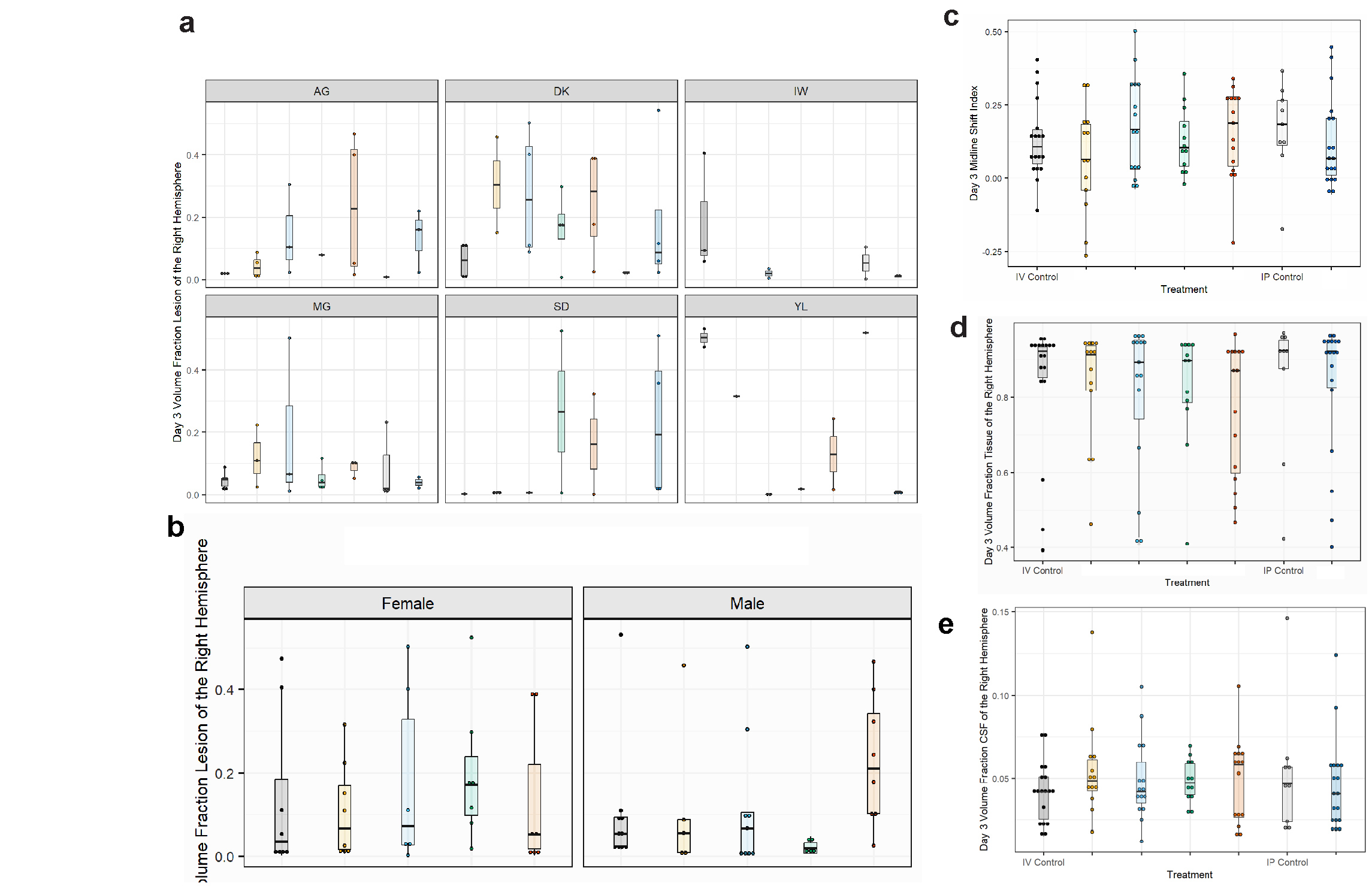

Supplement: Supplementary file 1 — Supplementary Material 1 (JPG 452 KB) [file 12975_2025_1407_MOESM1_ESM.jpg]
